# Antioxidant Capacities and Phenolic Levels of Different Varieties of Serbian White Wines 

**DOI:** 10.3390/molecules15032016

**Published:** 2010-03-22

**Authors:** Milan N. Mitić, Mirjana V. Obradović, Zora B. Grahovac, Aleksandra N. Pavlović

**Affiliations:** Faculty of Sciences and Mathematics, Department of Chemistry, University of Niš, Višegradska 33, P.O.Box 224, 18000 Niš, Serbia; E-Mails: milanmitic83@yahoo.com (M.N.M.); obradovic.mirjana@yahoo.com (M.V.O.); grahovacz@yahoo.com (Z.B.G.)

**Keywords:** white wines, total phenolics, total antioxidant activity, flavonoids, hydroxycinnamic acids

## Abstract

The biologically active compounds in wine, especially phenolics, are responsible for reduced risk of developing chronic diseases (cardiovascular disrease, cancer, diabetes, *etc*.), due to their antioxidant activities. We determined the contents of total phenolics (TP) and total flavonoids (TF) in selected Serbian white wines by colorimetric methods. Total antioxidant activity (TAA) of the white wines was analyzed using the 2,2-diphenyl-1-picrylhydrazyl (DPPH) radical scavenging capacity assay. Međaš beli had the highest content of TP, TF and TAA. The radical scavenging capacity (RSC) and total antioxidant activity (TAA) of white wines were 15.30% and 1.055 mM Trolox equivalent, respectively. Total phenolic (TP) and total flavonoid (TF) contents in white wines ranged from 238.3 to 420.6 mg gallic acid equivalent per L of wines and 42.64 to 81.32 mg catechin equivalent per L of wines, respectively. A high and significant correlation between antioxidant activity and total phenolic content was determined in wines (R^2 ^= 0.968, p < 0.01). For the individual polyphenols determination we used a high performance liquid chromatography (HPLC)-diode array detection (DAD) technique. The majority of white wine polyphenols was represent by four hydroxycinnamic acids (HCAs).

## 1. Introduction

Wine is a widely consumed beverage with thousands of years of tradition. The quality of a wine depends on its numerous constituents, with the presence/absence and amount of a given chemical playing a considerable role. Phenolic compounds are always present and they contribute markedly to the color, flavor, bitterness and astringency of the final product. In addition to their direct role, phenolic compounds may also contribute to the sensory and chemical qualities of wine because of their interaction with other compounds, namely proteins, polysaccharides or other polyphenols [1]. Moreover, interest in phenolic compounds in wine has increased in recent years because of their potential beneficial effects on human health [2].

The phenolic composition is deeply influenced by three sets of factors: the nature of the raw material (grape variety, its degree of maturation, the nature of the soil and the climate) [3], the vinification techniques [2,4] and evaluation of phenolic compounds during the ageing of wine [2]. White wines are usually made from the free running juice, without grape mash, having no contact with the grape skins. This was thought to be the main reason for relatively low polyphenol content and thus for the lower antioxidant activity of white wine in comparison to red wine.

The production of white wines involves a great effort to avoid extensive contact with oxygen, which might be deleterious in terse of color alteration (browning) and eventually determination of the overall quality and marketability. Browning in wines is the result of a complex series of oxidation reactions that take place during processing, ageing and storage, which give rise to a brown color that increases color intensity, decreases brightness and raises the browning index [5]. The most important polyphenolic constituents in white wines, both in term of quantity and ability to participate in redox reactions, are the hydroxycinnamates and flavanols. In particular, oxidation of *ortho*-dihydroxyphenolic compounds such as (+)-catechin, (-)-epicatechin, caffeic and other hydroxy-cinnamic acids leads to the formation of yellow or brown products due to the polymerization of *ortho*-quinones [6]. Other constituents of the wine, such as transition metal ions, the presence of SO_2_ and ascorbic acid are of equal importance in polyphenol oxidation [7]. Sulphur dioxide and ascorbic acid, when added to wine, are able to reduce the *ortho-*quinones, while metal ions can catalyze the oxidation reactions [7].

Serbia, with 82,000 ha of vine area and around 2 million hL of annual wine productions, is one of the middle-range wine producers in Europe. It has different regions, each with their specific climates, yield and distinct wine varieties. The main wine growing areas all lay along rivers: the Danube, all three Moravas, the Timok or the Nišava. There are several indigenous wines like the pale Smederevka or Slankamenka and the rosé Prokupac and Župljanka, that bear the names of the towns or regions where they originated. The aim of this study was to characterize the phenol components and antioxidant profile of white wines produced in the Zapadnomoravski and Banat wine regions of Serbia.

## 2. Results and Discussion

### 2.1. Total phenolic and flavonoid contents

Total phenolic contents of the 10 tested nwhite wines are presented in Table 1. Of all the white wines analysed and produced in 2007, Međaš Beli and Terra Lazarica-Chardonnay had the highest total phenolic content (420.6 ± 12.0 and 358.0 ± 12.1 mg of gallic acid equivalents/L of wine, respectively), followed by Banatski Riesling, Graševina, Chardonnay, Muscat Ottonel and Smederevka. The phenolic content of Međaš Beli was significantly different from the others (p < 0.05). Significant differences were found in total phenolic content when comparing between Terra Lazarica-Chardonnay and Graševina and Banatski Riesling and Muscat Ottonel (p < 0.05); however, no significant differences in total phenolic content were found between Graševina and Muscat Ottonel, between Terra Lazarica-Chardonnay and Banatski Riesling, among Chardonnay, Muscat Ottonel and Smederevka. Significant differences were also found in comparisons between Zupski Riesling and Car Constantin-Semillon, which were produced in 2003. In this study, there was a 1.76-fold difference in total phenolic content between the highest and lowest ranked varieties, Međaš Beli and Smederevka (p < 0.05). It is well known that genetic and agronomic or environmental factors play important roles in phenolic composition and thus nutritional quality of crops.

**Table 1:** Total phenol (TP) and total flavonoid (TF) contents.

Wine and vintage	TP^a^	TF^b^	TF/TP
	(mg GAE/L)	(mg CE/L)	
Zapadnomoravski wine region			
			
Graševina (2007)	268.8 ± 8.0 b	46.50 ± 1.16 a	0.17
Smederevka (2007)	238.3 ± 6.6 a	45.30 ± 1.29 a	0.19
Chardonnay (2007)	253.8 ± 7.8 ab	46.29 ± 1.43 a	0.18
Terra Lazarica-Chardonnay (2007)	358.0 ± 10.2 dc	65.00 ± 1.86 c	0.18
Međaš beli (2007)	420.6 ±12.0 e	81.32 ± 2.36 d	0.19
Župski Riesling (2003)	380.0 ± 12.1 d	63.99 ± 1.92 c	0.17
Car Konstantin-Semillon (2003)	261.5 ± 7.8 ab	48.25 ± 1.38 a	0.18
			
Banat wine region			
			
Traminac (2004)	270.2 ± 7.9 b	50.02 ± 1.55 a	0.18
Banatski Riesling (2007)	330.2 ± 9.4 c	56.29 ± 1.66 b	0.17
Muscat Ottonel (2007)	252.0 ± 7.5 ab	47.88 ± 1.35 a	0.18

^a^ The level of total phenolics is expressed as gallic acid equivalent (GAE) and data are reported as mean ± standard deviation (n = 3); ^b ^The level of total flavonoids is expressed as catechin equivalent (CE) and data are reported as mean ± standard deviation (n = 3); ^c-e ^Bars with no letters in common are significantly different (p < 0.05) in the same column.

### 2.2. Total flavonoid content

Total flavonoids of the 10 white wines were measured (Table 2). The Međaš Beli presented the highest flavonoid content (81.32 ± 2.36 mg of catechin equivalents/L of wines, (p < 0.05), followed by Terra Lazarica-Chardonnay, Župski Riesling, Banatski Riesling, Traminac, Car Konstantin-Semillon, Muscat Ottonel, Graševina, Chardonnay and Smederevka. The flavonoid content of Međaš Beli was significantly different from each others (p < 0.05) (except for Terra Lazarica-Chardonnay). Significant differences were found in total flavonoid content in comparisons between Banatski Riesling and Župski Riesling, Chardonnay and Terra Lazarica-Chardonnay, Graševina and Banatski Riesling and Terra Lazarica-Chardonnay and Muscat Ottonel. No significant differences in the total flavonoid content were found in comparisons between Terra Lazarica-Chardonnay and Župski Riesling, and among Traminac, Car Konstantin-Semillon, Muscat Ottonel, Graševina, Chardonnay and Smederevka (p < 0.05). An approximately 1.79-fold difference in total flavonoid content was found between the highst and lowest ranked varieties, Međaš Beli and Smederevka (p < 0.05). The average percent amount of flavonoids in total phenolics was 18 (16% in Croatian white wines) [8]. The mean concentration of the phenolic content of the Serbian white wines was 303.3 mg GAE/L. This is higher than the levels quoted in the literature for white wines grown in different countries (see Table 2).

**Table 2:** Published values of phenolic content in different white wines.

Country	TP (mg GAE/L)	Reference
Croatia	161–431 (n = 15)	[8]
Croatia	301–402 (n = 4)	[9]
Croatia	231–273 (n = 2)	[25]
Croatia	191–652 (n = 3)	[26]
Greece	162–286 (n = 12)	[5]
Greece	213–277 (n = 5)	[27]
Greece	267 (n = 1)	[13]
Spain	89–407 (n = 17)	[10]
Spain	178–293(n = 5)	[28]
Italy	170–260 (n = 16)	[29]
Italy	96–146 (n = 3)	[30]
Chech Republic	103–125 (n = 7)	[31]
South Africa	242–292 (n = 4)	[13]

### 2.3. Total antioxidant activity of wines using the DPPH scavenging assays

The radical scavenging capacity was evaluated by measuring the scavenging activity of examined wine samples on 2,2-diphenyl-1-picrylhydrazyl (DPPH) radicals. All antioxidant results are summarized in Table 3. A high phenolic content in the investigated white wines contributes to their increased antioxidant activity. The investigated white wines showed antioxidant behavior in the range from 19.05% to 12.7%. The percentage for Croatian white wines was 16.16–10.7 [8].

**Figure 1:**
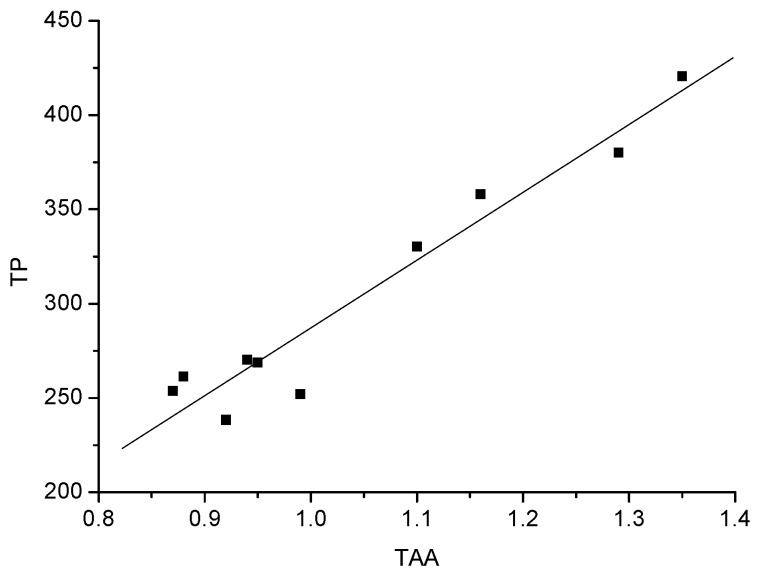
Correlation between total phenolics and total antioxidant activity.

**Figure 2:**
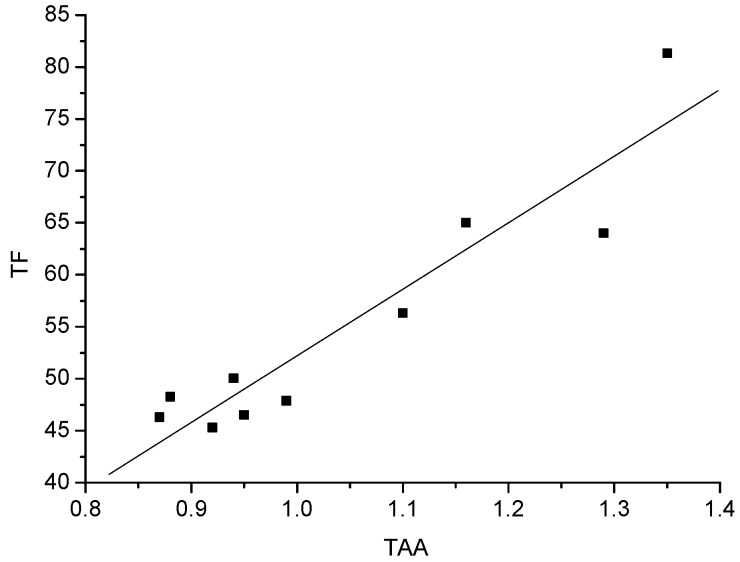
Correlation between total flavonoids and total antioxidant activity.

Total antioxidant activity (TAA) of the 10 white wines, expressed as millimoles (mmol) of Trolox equivalents per L of wine, is shown in Table 3. Međaš beli had the highest antioxidant activity, followed by Župski Riesling, Terra Lazarica-Chardonnay, Banatski Riesling, Muscat Ottonel, Graševina, Traminac, Smederevka, Car Konstantin-Semillon and Chardonnay (p < 0.05). A significant difference was found among Međaš beli, Terra Lazarica-Chardonnay and Chardonnay. The total antioxidant activities of Banatski Riesling and Muscat Ottonel were similar (p < 0.05), but lower (p < 0.05) than that of Međaš beli. No significant difference (p < 0.05) was found among Muscat Ottonel, Graševina, Traminac, Smederevka, Car Konstantin-Semillon and Chardonnay. The varieties containing high total phenolic contents had higher antioxidant activities. The present study reveals a very good correlation between total antioxidant activity and total phenolics (Figure 1) (R^2 ^= 0.968). Also, the significant correlation between the total flavonoid content, and antioxidant activity (R^2 ^= 0.938) of the tested white wine samples was confirmed (Figure 2). 

**Table 3:** Radical scavenging capacity (RSC) and total antioxidant activity (TAA) in different white wines.

Wine and vintage	RSC (%)	TAA^a^ (mM TE/L)
Zapadnomoravski wine region		
Graševina (2007)	14.00	0.95 ± 0.02^ a^^,b^
Smederevka (2007)	13.50	0.92 ± 0.03 ^a^
Chardonnay (2007)	12.73	0.87 ± 0.03 ^a^
Terra Lazarica-Chardonnay (2007)	16.30	1.16 ± 0.05 ^c^^,d^
Međaš beli (2007)	19.05	1.35 ± 0.06 ^e^
Župski Riesling (2003)	18.64	1.29 ± 0.03 ^d^^,e^
Car Konstantin-Semillon (2003)	12.83	0.88 ± 0.03 ^a^
Banat wine region		
Traminac (2004)	13.82	0.94 ± 0.05 ^a^
Banatski Riesling (2007)	16.04	1.10 ± 0.04 ^b^^,c^
Muscat Ottonel (2007)	14.95	0.99 ± 0.06 ^a^^,b^

^a^ The level of total antioxidant activity is expressed as Trolox equivalent (TE) and data are reported as mean ± standard deviation (n = 3).^c-e^ Bars with no letters in common are significantly different (p < 0.05) in the same column.

TAA values of the Serbian white wines analized in this study were in the range obtained for white wines from other wine-producing countries such as Spain (0.30–2.68 mM TE/L) [14] and 0.14–1.45 mM TE/L) [20] Portugal (1.4–2.9 mM TE/L) [21] and south African (0.54–0.72 mM TE/L) [19]. 

### 2.4. HPLC analysis

Tartaric esters of hydroxycinnamic acids represent the main non-flavonoid polyphenols in white grapes, mainly concentrated in the grapes fresh and thus in the wines made from white varieties. They represent 80% of all polyphenols of white grape juice [22]. They are involved in the browning reaction of must and wine, are precursors of volatile phenols and have antimicrobial and antioxidant activity. Their antioxidant properties may exert a positive health effect that is attributed to moderate wine consumption. 

Hydroxycinnamic acids and their tartaric acid derivatives have been monitored at 320 nm, since that is their characteristic wavelength, together with GRP (grape reaction product or 2-S-glutathionyl-t-caftaric acid).GRP is the product of the reaction between caftaric acid and glutation, which, as can be observed, is only detectable in the wine and not in the grape skins [23].

**Figure 3:**
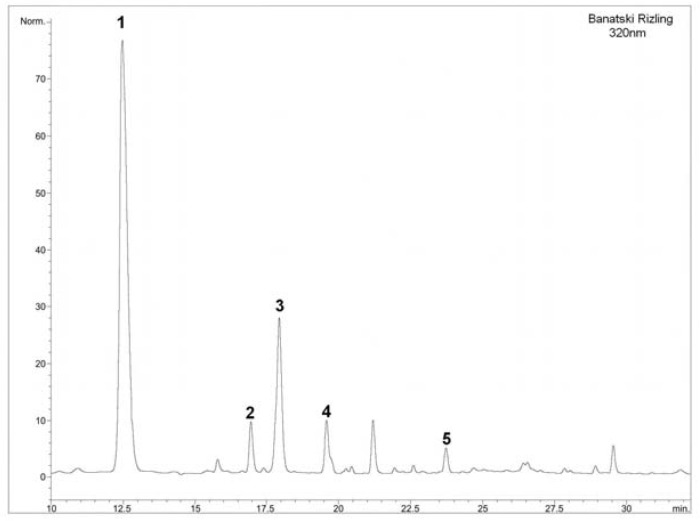
Chromatogram of a white wine. Identification of compounds: t-caftaric acid (1), GRP (2), *trans*-coutaric acid (3), caffeic acid (4), *p*-coumaric acid (5).

The chromatographic of some white wines monitored at 320 nm is given in Figure 3. The quantification of results for hydroxycinnamic acids in the analyzed samples is shown in Table 4. The predominant hydroxycinnamic acid was caftaric, followed by coutaric, caffeic and coumaric acid (Chardonny, 2007; Banatski Riesling, 2007; Graševina, 2007). The ratio of *trans*-caftaric acid (to all measured hydroxycinnamic acids) from Chardonnay, Banatski Riesling and Grasevina was on average 76.0%. Caftaric acid plays an important role in phenol oxidation and oxidative browning in must. The oxidized derivatives of coutaric and caftaric acid provide the yellowish-gold color in white wine. The values are within the range reported for white wines by other researchers [24,25,26,27]. 

**Table 4:** Hydroxycinnamic acids (mg/L) of white wines.

	Chardonnay	Banatski Riesling	Graševina	Traminac	Semillon
	(2007)	(2007)	(2007)	(2004)	(2003)
Caffeic acid	1.57 ± 0.18	3.39 ± 0.65	2.27 ± 0.82	7.70 ± 0.98	3.88 ± 0.80
*p*-coumaric acid	nd	0.62 ± 0.09	0.71 ± 0.08	2.02 ± 0.65	1.72 ± 0.06
*trans*-caftaric acid	14.71 ± 1.1	43.53 ± 1.5	21.96 ± 1.2	7.56 ± 0.85	nd
*trans*-coutaric acid	2.66 ± 0.74	5.74 ± 0.76	3.71 ± 0.90	1.36 ± 0.53	1.21 ± 0.22
GRP	1.13 ± 0.15	2.39 ± 0.35	nd	1.91 ± 0.48	4.14 ± 0.52

The data are reported as mean ± standard deviation (n = 3).

### 2.5. Principal component analysis

**Figure 4:**
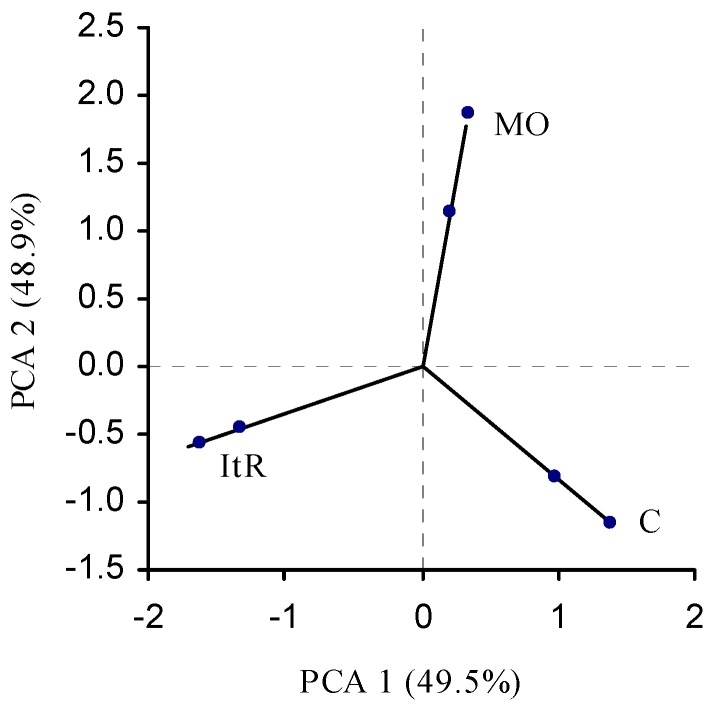
Principal component score plot (PC1 and PC2) of the studied white wines based on spectrophotometric data for the total phenols.

Principal component analysis (PCA) was applied in order to investigate the possible grouping of white wines (2007 vintage). From Table 5 and the correlation score plot in Figure 4, it can be seen that the first principal component (PC1) accounting for 49.544% of the variability and the second principal component (PC2) accounted for 48.853% of the variability, together PC1 and PC2 account for 98.397% of the total variance.

White wines from Muscat Ottonel (MO) grapes (Medjas Beli and Muscat Ottonel) are placed in the lower positive part of PC1, but high in PC2, while the white wines from Chardonnay (C) grapes (Chardonnay and Terra Lazarica-Chardonnay) are distributed in high positive part of PC1. White wines from Italian Riesling (ItR) grapes (Grasevina and Banatski Riesling) have a large negative contribution to PC1 and small negative contribution to PC2. This results suggest that the content of the phenolics in wine dependence on the grape varieties.

**Table 5:** Explained variance and eigenvalues (PC1: first principal component, PC2: second principal component, PC3: third principal component).

Value	PC1	PC2	PC3
Eigenvalue	1.486	1.330	0.048
% of variance	49.544	48.853	1.603
Cumulative %	49.544	98.397	100.000

## 3. Experimental

### 3.1. Wine samples

10 commercially available wines made from different grape varieties were selected and then analyzed (June, 2009). A list of all wines analyzed in this study with their geographical origin and type is presented in Table 6. All samples were stored at 10 °C in the dark and analyzed shortly after opening. All wines analyzed are frequently consumed in Serbia.

**Table 6:** Compositional factors determined in the white wines.

Wine and vintage	Grape varieties	Alcohol Vol. (%)	Acidity ^a^	pH
Zapadnomoravski wine region				
Graševina (2007)	Italien Riesling	11.5	6.93	3.57
Smederevka (2007)	Smederevka	10.5	6.83	3.50
Chardonay (2007)	Chardonnay	11.0	6.70	3.40
Terra Lazarica-Chardonnay (2007)	Chardonnay	12.0	6.72	3.42
Međaš beli (2007)	Chardonnay, Sauvignon, Muscat Ottonel	11.5	6.38	3.57
Župski Riesling (2003)	Italian Rieling	10.7	6.12	3.81
Car Konstantin-Semillon (2003)	Semillon	11.5	6.35	3.62
				
Banat wine region				
Traminac (2004)	Traminer	12.5	6.30	3.24
Banatski Riesling (2007)	Italian Riesling, Smederevka, Župljanka, Kreazer	11.3	5.98	3.22
Muscat Ottonel (2007)	Muscat Ottonel	11.5	6.42	3.38

^a^ Titritable acidity expressed as grams per liter tartaric acid.

### 3.2. Chemicals

All chemicals and reagents were of analytical grade and were obtained from Sigma Chemical Co. (St. Louis, MO, USA), Aldrich Chemical Co. (Steineheim, Germany), and Merck (Darmstadt, Germany). Calibration curves were obtained from triplicate injections of five concentrations.

### 3.3. Determination of total phenolic content (TP)

The total phenol content of the wines was measured spectrophotometrically at 760 nm after the reaction with Folin-Ciocalteu phenol reagent, according to the manual method described by Singleton *et al.* [28] using gallic acid as a calibration standard. Results are given as gallic acid equivalents (GAE, mg/L). All measurements were performed in triplicate.

### 3.4. Determination of total flavonoid content (TF)

The total flavonoid content in selected wine samples was determined spectrophotometrically [29], using a method based on the formation of complex flavonoid-aluminium. The absorbance was measured at 510 nm. The concentration of the total flavonoid compounds in the wines was expressed as catechin equivalent (mg/L). The results in every essay were obtained from three parallel determinations.

### 3.5. Measurement of the DPPH scavenging activity

The free radical scavenging activity of white wines was determined according to the method of Liu *et al.* [30]. 0.1 mL white wine sample (at the dilution of 1:10 with methanol) were added to 1.9 mL of a 0.1 mM methanolic solution of DPPH. The absorbance at 517 nm was measured after the solution had been allowed to stand in the dark for 30 min. Radical scavenging capacity (RSC), expressed as a percentage, was calculate by using the formula:

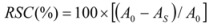

where *A_0_* is the absorbance of the blank sample and *A_s_* is the absorbance of the tested white wine sample. Using Trolox as a standard, total antioxidant activity (TAA) was expressed as milimoles of Trolox equivalents (TE) per L of white wine. 

### 3.6. HPLC analysis of hydroxycinnamic acids

For determination of the hydroxycinnamic acids we used an HPLC Agilent-1200 series instrument equipped with a UV-Vis photodiode array (DAD) detector. The column was termostated at 30 °C. After injecting 5 µL of sample, separation was performed in an Agilent-Eclipse XDB C-18 4.6x150 mm column. Two solvents were used for the gradient elution: A-(H_2_O + 5%HCOOH) and B-(80%ACN + 5%HCOOH + H_2_O). The elution program used was as follows: from 0 to 28 min, 0.0% B, from 28 to 35 min, 25% B, from 35 to 40 min, 50% B, from 40 to 45 min, 80% B, and finally for last 10 min again 0% B. The different hydroxycinnamic acids analysed were tentatively identified according to their order of elution, retention times of pure compounds (caffeic, coumaric and ferulic acids) and the characteristics of the UV-Vis spectra published in different studies [23,31,32]. The calibration curves were obtained by injecting standards with different concentrations. The range of the linear calibration curves (R^2^_1,2,3 _> 0.97) was 1.0 to 100.0 mg/L. Quantification of non-commercial compound was made using the calibration curves belonging to the most similar compound: caffeic acid for *cis*- and *trans*-caftaric acids and GRP; *p*-coumaric acid for *cis*- and *trans*-coutaric acids and ferulic acid for *cis*- and *trans*-fertaric acids. The calibration graphs were constructed for every standard by plotting each chromatographic peak area against its corresponding concentration.

### 3.7. Statistical analysis

Experiments were reported at least three times, and the data were analyzed statistically. All results are given as mean ± standard deviation (SD). Analysis of variance ANOVA tests were established for total phenolics, flavonoids and antioxidant activity in white wines. 

## 4. Conclusions

This study shows that although all the analyzed white wines contained phenolic compounds and flavonoids, their contents are markedly different among grape varieties. All white wines varieties represent a potential source of natural antioxidants, but the Međaš Beli variety showed a better performance for wines which were produced in 2007. Also, the present data indicated differences in phenolic composition and antioxidant activity of different cultivar wines in spite of differences in agronomic factors and climatic conditions. In addition the total antioxidant activity of wines, correlated to their phenolic contents, but some individual phenilic compounds had higher antioxidant activity then others. Because of that, future research should include investigation of the effect of different individual phenolic compound on the antioxidant activity of wines.
